# Varicella – trend and challenge for surveillance and for introduction of routine immunization in Romania

**DOI:** 10.1186/1471-2334-14-S7-P77

**Published:** 2014-10-15

**Authors:** Alexandru Rafila, Alina Zaharia, Ruxandra Ioana Ștefan, Aurora Stănescu, Adriana Pistol, Daniela Pițigoi

**Affiliations:** 1 Carol Davila University of Medicine and Pharmacy, Bucharest, Romania; 2 National Institute for Infectious Diseases "Prof. Dr. Matei Balş", Bucharest, Romania; 3 National Institute of Public Health, Bucharest, Romania

## Background

Varicella is a viral disease which can be easily prevented through vaccination. In European Union the surveillance systems for varicella and herpes zoster are highly heterogeneous or absent and seventeen countries have recommendations on varicella vaccination. In Romania varicella vaccination is not included in the National Immunization Program (NIP), although a major number of cases are reported annually. Our aim is to provide an overview of surveillance system and epidemiological issues of varicella in Romania in the last 10 years (2004-2013).

## Methods

In Romania there is no specific surveillance system for varicella, but there is a quarterly mandatory notification system of clinical confirmed cases and deaths, by age groups and place of residence. The system covers the total country population. We conducted a retrospective study using information reported by general practitioners and hospitals to the National Statistics Centre and National Centre for Surveillance and Control of Communicable Diseases.

## Results

A total of 504,844 varicella cases were reported in Romania from 2004 to 2013 and 2 deaths (young adults with severe pneumonia related to varicella). The mean annual incidence 2004-2013 was 238.2 cases per 100,000 inhabitants, with the highest value from European Union in 2007 (326.9 cases per 100,000 inhabitants). Most of the cases (73.6%) live in urban areas. The most affected age-group were children 5-9 years (mean annual incidence 2004-2013: 1362.7 cases per 100,000 inhabitants), followed by 1-4 years (1297.2 cases per 100,000 inhabitants) and 10-14 years (947.2 cases per 100,000 inhabitants).

## Conclusion

Varicella is a very common communicable disease in Romania. The current system of notification does not collect information regarding the clinical aspects, severity and real impact of the disease. For these reasons is important to organize a case-based surveillance system (e.g., sentinel surveillance system at the beginning) and to assess the opportunity to introduce the routine varicella vaccination into NIP in Romania.

